# Prenatal Organophosphorus Pesticide Exposure and Child Neurodevelopment at 24 Months: An Analysis of Four Birth Cohorts

**DOI:** 10.1289/ehp.1409474

**Published:** 2015-09-29

**Authors:** Stephanie M. Engel, Asa Bradman, Mary S. Wolff, Virginia A. Rauh, Kim G. Harley, Jenny H. Yang, Lori A. Hoepner, Dana Boyd Barr, Kimberly Yolton, Michelle G. Vedar, Yingying Xu, Richard W. Hornung, James G. Wetmur, Jia Chen, Nina T. Holland, Frederica P. Perera, Robin M. Whyatt, Bruce P. Lanphear, Brenda Eskenazi

**Affiliations:** 1 Department of Epidemiology, UNC Gillings School of Global Public Health, Chapel Hill, North Carolina, USA; 2 Center for Children’s Environmental Health, School of Public Health, University of California, Berkeley, Berkeley, California, USA; 3 Department of Preventive Medicine, Icahn School of Medicine at Mount Sinai, New York, New York, USA; 4 Department of Population and Family Health, Mailman School of Public Health, Columbia University, New York, New York, USA; 5 Department of Biostatistics, UNC Gillings School of Global Public Health, Chapel Hill, North Carolina, USA; 6 Department of Environmental Health Sciences, Columbia Center for Children’s Environmental Health, Mailman School of Public Health, Columbia University, New York, New York, USA; 7 Department of Environmental Health, Rollins School of Public Health, Emory University, Atlanta, Georgia, USA; 8 Division of General and Community Pediatrics, Cincinnati Children’s Hospital Medical Center, Cincinnati, Ohio, USA; 9 Department of Microbiology, Icahn School of Medicine at Mount Sinai, New York, New York, USA; 10 Simon Fraser University, Vancouver, British Columbia, Canada

## Abstract

**Background:**

Organophosphorus pesticides (OPs) are used in agriculture worldwide. Residential use was common in the United States before 2001.

**Objectives:**

We conducted a pooled analysis of four birth cohorts (children’s centers; n = 936) to evaluate associations of prenatal exposure to OPs with child development at 24 months.

**Methods:**

Using general linear models, we computed site-specific and pooled estimates of the association of total dialkyl (ΣDAP), diethyl (ΣDEP), and dimethylphosphate (ΣDMP) metabolite concentrations in maternal prenatal urine with mental and psychomotor development indices (MDI/PDI) and evaluated heterogeneity by children’s center, race/ethnicity, and PON1 genotype.

**Results:**

There was significant heterogeneity in the center-specific estimates of association for ΣDAP and ΣDMP and the MDI (p = 0.09, and p = 0.05, respectively), as well as heterogeneity in the race/ethnicity-specific estimates for ΣDAP (p = 0.06) and ΣDMP (p = 0.02) and the MDI. Strong MDI associations in the CHAMACOS population per 10-fold increase in ΣDAP (β = –4.17; 95% CI: –7.00, –1.33) and ΣDMP (β = –3.64; 95% CI: –5.97, –1.32) were influential, as were associations among Hispanics (β per 10-fold increase in ΣDAP = –2.91; 95% CI: –4.71, –1.12). We generally found stronger negative associations of ΣDAP and ΣDEP with the 24-month MDI for carriers of the 192Q PON1 allele, particularly among blacks and Hispanics.

**Conclusions:**

Data pooling was complicated by center-related differences in subject characteristics, eligibility, and changes in regulations governing residential use of OPs during the study periods. Pooled summary estimates of prenatal exposure to OPs and neurodevelopment should be interpreted with caution because of significant heterogeneity in associations by center, race/ethnicity, and PON1 genotype. Subgroups with unique exposure profiles or susceptibilities may be at higher risk for adverse neurodevelopment following prenatal exposure.

**Citation:**

Engel SM, Bradman A, Wolff MS, Rauh VA, Harley KG, Yang JH, Hoepner LA, Barr DB, Yolton K, Vedar MG, Xu Y, Hornung RW, Wetmur JG, Chen J, Holland NT, Perera FP, Whyatt RM, Lanphear BP, Eskenazi B. 2016. Prenatal organophosphorus pesticide exposure and child neurodevelopment at 24 months: an analysis of four birth cohorts. Environ Health Perspect 124:822–830; http://dx.doi.org/10.1289/ehp.1409474

## Introduction

Organophosphorus pesticides (OPs) are a class of insecticides that act by irreversibly inactivating acetylcholinesterase, causing a build-up of acetylcholine in the synapse that leads to uncontrolled activation of sodium ion channel receptors (Fukuto 1990). Acute OP toxicity results from this acetylcholinesterase inhibition; however, biological effects of chronic low-dose exposure may not be mediated by this pathway (Ray and Richards 2001), and these alternate mechanisms have been shown to result in persistent behavioral and/or neurocognitive deficits in animals (Icenogle et al. 2004; Levin et al. 2002; Ricceri et al. 2003; Timofeeva et al. 2008; Vatanparast et al. 2013). Paraoxonase 1 (PON1), an antioxidant, is a key enzyme that deactivates certain OPs (Costa et al. 2003). The first metabolism step, mediated primarily by P450 enzymes, converts the organothiophosphate to an oxon, the biologically active form, which PON1 then hydrolizes (Costa et al. 2003). Some functional polymorphisms in *PON1* have been extensively studied and correlate strongly with enzyme expression levels (–108 C/T, T having reduced enzyme expression) or substrate-specific catalytic efficiency (Q192R, Q having slower catalytic efficiency for the chlorpyrifos oxon) (Costa et al. 2003).

Before 2001, residential applications of OPs to control pests were common in the United States particularly in urban, multi-housing units. In July 2000, the Environmental Protection Agency (EPA) and Dow Chemical agreed to eliminate all residential use of chlorpyrifos in the United States by 31 December 2001. In December 2000, the U.S. EPA and registrants agreed to phase out and eliminate all indoor residential uses of diazinon by 31 December 2002 (U.S. EPA 2000, 2001). Diet from conventionally grown produce continues to be an important route of exposure in the general population to these and other OPs. Although OP pesticides continue to be widely used in agriculture, their usage has decreased over the last few decades—from 225 million pounds in 1980 to < 100 million pounds in 2007 (Grube et al. 2011); this decline is consistent with biomonitoring data, which show a decline in median urinary OP metabolite levels over a 6-year period in a representative sample of the U.S. general population (Barr et al. 2011). Nonetheless, residents in agricultural areas can be exposed if employed in agriculture, or by take-home exposure on clothes or shoes from household agricultural workers and by pesticide drift (Curl et al. 2002).

Starting in 1998, the National Institute of Environmental Health Sciences (NIEHS) and EPA jointly funded multiple Children’s Environmental Health and Disease Prevention Research Centers across the United States. Four Centers pursued research on prenatal exposure to OP insecticides: Columbia University (“Columbia”), Mount Sinai Medical Center (“Mount Sinai”), University of California at Berkeley CHAMACOS study (“CHAMACOS”), all starting in 1998, and Cincinnati Children’s Hospital HOME Study (“HOME”) starting in 2003. These four centers targeted urban (Mount Sinai and Columbia), urban and suburban (HOME), and rural/agricultural (CHAMACOS) populations, of varying sociodemographic and racial/ethnic characteristics. Uniform biomarkers of exposure and metabolism and neurodevelopmental assessments were employed. Because individual centers had limited power to explore associations within susceptible subgroups, we describe a pooled analysis of these four cohorts in relation to the mental and psychomotor development of children 24 months of age, taking into account both genetic and demographic susceptibility factors.

## Methods

The enrollment criteria and procedures used by each center are described below and summarized in Table 1:

**Table 1:** Summary of study design characteristics by cohort.

Subject characteristics	UC Berkeley Center for Environmental Research and Children’s Health CHAMACOS Cohort	Cincinnati Children’s Environmental Health Center HOME Study	Columbia Center for Children’s Environmental Health Cohort	Mount Sinai Children’s Environmental Health and Disease Prevention Research Center Cohort
Study location & source population	Agricultural population in the Salinas Valley, California	Urban and suburban population residing in Brown, Butler, Clermont, Hamilton, or Warren counties in Ohio	Urban population residing in South Bronx or Northern Manhattan in New York City	Urban population consisting of women receiving antenatal care at the Mount Sinai Hospital or two private obstetric practices on the Upper East Side of Manhattan, New York City
Years of enrollment	1999–2000	2003–2006	2000–2001	1998–2002
Eligibility criteria	Receiving prenatal care at a designated clinic; < 20 weeks’ at enrollment; eligible for medical; 18 years or older; English or Spanish speaking	Living in housing built before 1978; < 19 weeks gestation at enrollment	African-American or Dominican women; 18–35 years of age; first prenatal care visit < 20 weeks gestation	Primiparous; English or Spanish speaking; first prenatal care visit < 26 weeks gestation.
Exclusion criteria	Twins; gestational or preexisting diabetes; hypertension; stillbirths; birth weight < 500 g	Twins; HIV positive; receiving seizure, thyroid, or chemotherapy/radiation medications	Twins; diabetes; hypertension; known HIV infection; smoking; Illicit drug use	Twins; diabetes, hypertension or thyroid diseases; Illicit drug use or alcohol abuse during pregnancy; severe pregnancy complications including very premature birth (< 32 weeks or < 1,500 g at delivery); congenital malformations
Exposure measurement	2 urine samples at 13 and 26 weeks gestation averaged on a creatinine-corrected basis	2 urine samples at 16 and 26 weeks gestation averaged on a creatinine-corrected basis	1 urine sample at 32 weeks gestation	1 urine sample at 31 weeks gestation

***Study populations.* The University of California, Berkeley, Center for Environmental Research and Children’s Health.** Between October 1999 and October 2000, the Center for the Health Assessment of Mothers and Children of Salinas (CHAMACOS) study enrolled 601 pregnant women living in the Salinas Valley of California and receiving prenatal care at the Natividad Medical Center or one of five prenatal care clinics serving farmworker populations. Eligible women were < 20 weeks’ gestation at enrollment, were eligible for Medi-Cal, were ≥ 18 years of age, spoke English or Spanish, and planned to deliver at the Natividad Medical Center. Prenatal maternal spot urine specimens were obtained at enrollment on average at 13 weeks gestation and again at 26 weeks gestation. There were 538 infants after subtracting losses due to miscarriage, moving, or dropping from the study before delivery. Subsequent exclusions included gestational or preexisting diabetes, hypertension, twin births, stillbirths, and one very low birth weight infant (< 500 g), resulting in a final sample size of 488 pregnancies (Eskenazi et al. 2004). The Bayley Scales of Infant Development, 2nd Edition (BSID-II) was administered to children in Spanish and/or English by psychometricians who were unaware of the child’s exposure level (*n* = 372). The BSID-II is a standardized developmental test that assesses current developmental functioning and generates a Mental Development Index (MDI) and Psychomotor Developmental Index (PDI) (Bayley 1993). Together these describe the child’s current level of cognitive, language, and fine and gross motor development. Psychometricians were trained using standardized protocols and were supervised for quality assurance by a clinical neuropsychologist. Assessments were performed in a private room at the CHAMACOS research office or in a recreational vehicle modified to be a mobile testing facility (Eskenazi et al. 2007).

**Cincinnati Children’s Environmental Health Center HOME Study.** The Health Outcomes and Measures of the Environment (HOME) Study enrolled a total of 468 women between March 2003 and January 2006. Because the HOME Study contains a nested, randomized trial of in-home lead and injury hazard controls, women had to have been living in housing built before 1978. Additional eligibility criteria included < 19 weeks gestation upon enrollment; living in Brown, Butler, Clermont, Hamilton, or Warren counties in Ohio; intention to continue prenatal care and deliver at collaborating obstetric practices; HIV negative; and not receiving seizure, thyroid, or chemotherapy/radiation medications. From these women, prenatal urine specimens were obtained at approximately 16 and 26 weeks gestation. A total of 389 women delivered live-born, singleton infants without birth defects. The BSID-II was administered to 236 of these children at 24 months in a clinic setting by one of three examiners who were trained to reliability, and recertified in administration and scoring every 6 months. Examiners were blind to the exposure characteristics of the children (Chen A et al. 2014).

**The Columbia Center for Children’s Environmental Health.** The Columbia study enrolled 727 pregnant women between 1998 and 2006. The cohort was restricted to nonsmoking women 18–35 years old who self-identified as either African-American race or Dominican ethnicity, and who had resided in Northern Manhattan or the South Bronx in New York City for > 1 year before pregnancy. Women were excluded if they reported illicit drug use; had diabetes, hypertension, or known HIV; or had their first prenatal visit after gestational week 20. Women were considered fully enrolled if a 48-hr personal air sample and a blood sample was obtained either from the mother during her third trimester or from cord blood at delivery. The Columbia study used a blood-based biomarker of chlorpyrifos as the primary dosimeter of exposure in their population (Whyatt et al. 2004); however, from a subset of 91 women enrolled in 2000 and 2001, a maternal urine sample was obtained in the late third trimester (32.4 weeks; SD = 3.0) and analyzed for urinary OP biomarkers. Among these 91 women, the BSID-II was administered to 63 children in a study office by one of five trained bilingual research assistants who were assessed for reliability and blinded to exposure status. Every effort was made to maximize reliability in scoring by using standardized training procedures and regular quality control (Rauh et al. 2006).

**The Mount Sinai Children’s Environmental Health and Disease Prevention Research Center (Mount Sinai).** The Mount Sinai study enrolled 479 primiparous women with singleton pregnancies from the Diagnosis and Treatment Center prenatal clinic and two adjacent private practices between 1998 and 2002. Women were excluded if they had their first prenatal visit after 26 weeks of gestation; had chronic diseases such as diabetes, hypertension, or thyroid disease or developed a serious pregnancy complication that could affect fetal growth and development; and consumed more than two alcoholic beverages per day or who used illegal drugs (Berkowitz et al. 2003). Seventy-five women were subsequently excluded because of medical complications, very premature births (< 32 weeks gestation or < 1,500 g), delivery of an infant with congenital malformations, inability to obtain biological specimens before delivery, change of residence, or refusal to continue participation (Engel et al. 2007). Prenatal maternal spot urine specimens were obtained in the third trimester (mean = 31.2 weeks, SD = 3.7). Birth data were available for 404 mother–infant pairs. The BSID-II was administered to 225 of these children in English or Spanish at the study office by a trained examiner who was unaware of their exposure level and was supervised by a developmental psychologist. All examinations were scored by two independent examiners, and any discrepancies were resolved by discussion and review of materials (Engel et al. 2011).

***Biomarkers of OP exposure.*** For all studies, maternal urine samples were analyzed at the Centers for Disease Control and Prevention (CDC; Atlanta, GA) for six dialkylphosphate metabolites (including three diethylphosphate metabolites and three dimethylphosphate metabolites). CHAMACOS, Columbia, and Mount Sinai urine samples were analyzed at the CDC in 2002–2003, and those from the HOME study in 2010. Laboratory and quality control methods have been previously reported (Barr et al. 2005; Bravo et al. 2004). For the CHAMACOS and HOME studies where two prenatal urine samples were obtained, the average of the creatinine-corrected log_10_ urinary metabolite values was used as the best estimate of prenatal exposure. For the Mount Sinai and Columbia studies, only one prenatal urine sample was obtained. In cases where concentrations of individual metabolites were below the limit of detection (LOD) and an instrument-read value was not provided, a random value < LOD was imputed using maximum likelihood estimation based on a log-normal distribution truncated at the LOD. For samples in which a metabolite value was missing due to analytic interference (*n* = 23), the missing value within a class (diethyl or dimethyl) was imputed using the other non-missing values within that class, as has been previously described (Engel et al. 2007; Eskenazi et al. 2004). A complete description of the proportion of urines with biomarker values below the LOD or missing due to analytic interference is presented in Supplemental Material, Table S1. Diethyl and dimethylphosphate metabolites were then summed on a molar basis to obtain total diethylphosphate (ΣDEP) and total dimethylphosphate (ΣDMP) biomarker concentrations, respectively, which were subsequently summed to obtain total dialkylphosphate (ΣDAP) levels. Individual metabolites were corrected for urinary dilution by dividing by urinary creatinine concentrations before averaging (CHAMACOS and HOME) and then summing (all cohorts). Urine specimens that contained < 10 mg/dL of creatinine (*n* = 4) were not used in the analysis. Summed metabolite concentrations that were extremely low (e.g., the sum of three metabolites < LOD) were truncated at 3 SDs below the geometric mean to avoid including influentially low values.

**PON1 *genotyping.*** We used existing cord blood *PON1* –108C/T and Q192R polymorphism information from each of the centers. Although maternal *PON1* genotypes were available for some cohorts, they were not available for all. DNA extraction and genotyping methods have been previously described for the Mount Sinai (Chen J et al. 2003, 2005; Wetmur et al. 2005), CHAMACOS (Holland et al. 2006), and HOME populations (Rauch et al. 2012). For the Columbia cohort, child DNA was obtained from cord blood and genotyped for the –108C/T and Q192R polymorphism using TaqMan.

***Statistical analyses.*** Potential confounders were identified *a priori* after inspection of directed acyclic graphs (not shown) and included center (nominal categorical), maternal education (nominal categorical: < high school, high school, any college), marital status (nominal categorical: single vs. married/living with partner), and race/ethnicity (nominal categorical: white, black, Hispanic, other), smoking, alcohol or drug use during pregnancy (nominal categorical: ever/never), birth before 2001 (nominal categorical), and the quality of the home environment as assessed by the Home Observation for Measurement of the Environment score (categorical: center-specific quartiles) (Caldwell and Bradley 1984). Gestational age at delivery and birth weight were not evaluated for confounding because they are potentially causal intermediates. We additionally considered adjusting for examiner, language spoken in the home (English, Spanish, other), child sex, and any breastfeeding for at least 3 months (ever/never) if these variables improved the overall model precision. In comparing demographic and enrollment characteristics by center, it became clear that although these adjustment sets were desirable, they were not possible due to non-positivity (Cole and Hernán 2008). Non-positivity occurs when there is a lack of exposed and unexposed individuals at every combination of the values of the confounders under study (Cole and Hernán 2008; Westreich and Cole 2010). In this case, conflicting enrollment and exclusion and inclusion criteria resulted in centers with no participants in one or more levels of a potential confounders (e.g., race/ethnicity, smoking or drug use during pregnancy, birth before 2001, language spoken in the home, and Bayley examiner), which prevented us from implementing a fully adjusted model while simultaneously accounting for center. We therefore approached this analysis as follows: First we assessed center-related heterogeneity by including interaction terms for Center and ΣDAP/ΣDEP/ΣDMP in a model that included maternal education and marital status, alcohol use during pregnancy, child sex, Caldwell Home environment score, and breastfeeding for at least 3 months, using general linear models (SAS PROC GLM; SAS Institute Inc.). We also inspected the dose-response function of log_10_ ΣDAP and the MDI using restricted cubic splines to determine the best exposure variable form. Spline modeling was done using the RCS REG SAS Macro with knots at the 20%, 50%, and 80% quantiles (Desquilbet and Mariotti 2010). We selected the best exposure functional form by considering the significance of the spline terms using a 1-df (degree of freedom) Wald chi-square test (α = 0.05) (Desquilbet and Mariotti 2010), and the overall model Akaike information criterion (AIC) (favoring the model with the lowest AIC).

We next assessed heterogeneity by race/ethnicity using an expanded model which additionally included smoking and drug use during pregnancy, but not center, because center and race/ethnicity, smoking, and drug use during pregnancy could not be simultaneously adjusted for in a regression model due to non-overlapping distributions in these factors by center. We then assessed whether any race/ethnicity-specific associations varied by center by examining center-related heterogeneity within race/ethnicity groups.

Finally, to calculate an overall pooled association, we implemented linear mixed models in PROC MIXED, with a random intercept for center or race/ethnicity alternately. We additionally examined whether including Bayley examiner as a random effect along with center substantially improved the overall model fit. Finally, we assessed interactions with child *PON1* genotype (three-level categorical) overall and stratified by race/ethnicity (white, black, Hispanic). We considered all interactions to be significant at *p* < 0.10 using an *F*-test with 2 df. For main effects, we calculated 95% confidence intervals (CIs) and examined the magnitude of the point estimate, the width of the interval, as well as whether the interval crossed the null value. All analyses were conducted in SAS version 9.3. All subjects provided written informed consent at baseline and follow-up and institutional review board approval was obtained by the University of North Carolina at Chapel Hill, the Mount Sinai Medical Center, the Cincinnati Children’s Hospital Medical Center, the University of California at Berkeley Committee for the Protection of Human Subjects, and the Columbia University Medical Center.

## Results

***Participant characteristics by center.*** We present participant characteristics by center in Table 2. Maternal education was lowest among CHAMACOS participants, and highest among HOME participants. Hispanics made up the majority of participants from the CHAMACOS and Mount Sinai centers, although Mount Sinai also enrolled a substantial number of white and black participants. All Columbia participants reported black race, but 40% reported their country of origin as the Dominican Republic; these participants were classified as Hispanic in this analysis. The majority of HOME participants were white, although there were also black and Hispanic participants enrolled.

**Table 2:** Distribution of demographic characteristics in individual cohorts.

Subject characteristics	UC Berkeley CHAMACOS Cohort (*N *= 377)		Cincinnati Children’s HOME Study (*N *= 265)		Columbia University (*N *= 60)		Mount Sinai School of Medicine (*N *= 234)	
	*n*	Percent	*n*	Percent	*n*	Percent	*n*	Percent
Born before 2001	246	65.3	0	0	24	40	211	90.2
Maternal education
Less than high school	304	80.6	20	7.6	23	38.3	67	28.6
High school graduate/GED	36	9.6	26	9.8	20	33.3	48	20.5
Any college	37	9.8	219	82.6	17	28.3	119	50.8
Language spoken in home^*a*^
English	17	4.5	252	95.1	46	76.7	195	83.3
Any Spanish	356	94.4	0	0	10	16.7	38	16.2
Other	4	1.1	0	0	1	1.7	1	0.4
Maternal marital status^*b*^
Not married/not living with partner	65	17.2	40	15.1	45	75.0	113	48.3
Married/living with partner	312	82.8	225	84.9	15	25.0	121	51.7
Parity
Nulliparous	116	30.8	115	43.4	30	50.0	234	100.0
Parous	261	69.2	150	56.6	30	50.0	0	0
Maternal country of origin
Other	332	88.1	15	5.7	24	40.0	41	17.5
USA	45	11.9	250	94.3	36	60.0	162	69.2
Maternal race/ethnicity
White	3	0.8	190	71.7	0	0	51	21.8
Black	0	0	59	22.3	36	30.0	57	24.4
Hispanic	369	97.9	5	1.9	24	40.0	123	52.6
Other	5	1.3	11	4.2	0	0	3	1.3
Maternal age at delivery (years)^*c*^	377	26.5 (5.2)	265	29.9 (5.3)	60	24.7 (5.2)	234	23.8 (6.5)
Any alcohol use during pregnancy	90	23.9	125	47.2	9	15.0	29	12.4
Any illicit drug use during pregnancy	6	1.6	12	4.5	0	0	15	6.4
Any smoking during pregnancy	18	4.8	26	9.8	0	0	39	16.7
Child sex
Male	185	49.1	122	46.04	18	30.0	126	53.9
Female	192	50.9	143	53.96	42	70.0	108	46.2
Breastfeeding at 3 months	273	72.4	167	63.02	37	61.7	101	43.2
24-month Psychomotor Development Index (PDI)^*c*^	377	97.6 ± 10.5	264	91.1 ± 14.0	60	94.5 ± 9.4	228	93.6 ± 11.6
24-month Mental Development Index (MDI)^*c*^	377	85.8 ± 11.7	265	89.6 ± 14.3	60	84.4 ± 11.9	234	87.9 ± 13.8
^***a***^For Mount Sinai, primary language indicates the subject’s endorsement (*n *= 139), or when missing, the language in which the questionnaires were administered over the course of the study (*n *= 95). ^***b***^Columbia required “living with a partner” to include only those who cohabited for ≥ 7 years. ^***c***^Mean ± SD.								

**Figure 1:**
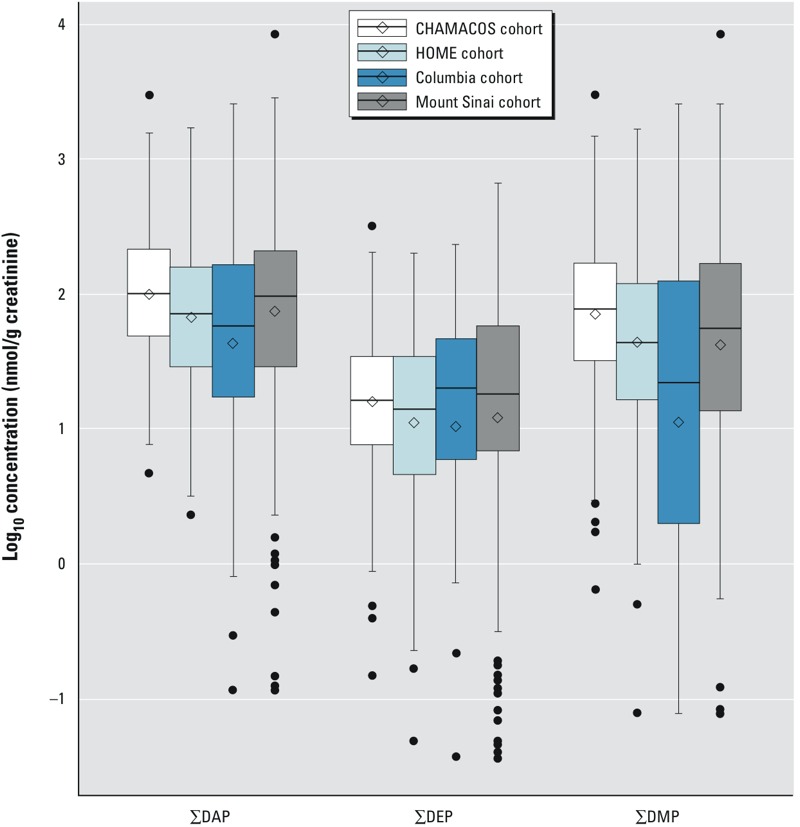
Distributions of dialkylphosphate metabolite sums in individual cohorts. The 25th, 50th, and 75th percentiles by center are represented by the lower, middle, and upper bars in the central boxes. The arithmetic mean by center is marked by the open diamond. Outliers at the upper and lower end of the exposure distribution are indicated by circles. Whiskers indicate 95% CIs.

***Exposure distributions by center.*** Prenatal urinary ΣDAP, ΣDEP, and ΣDMP biomarker concentrations can be found in Figure 1; see also Supplemental Material, Tables S1 and S2. Geometric mean ΣDAP and ΣDMP concentrations were substantially higher in the CHAMACOS cohort than in all other cohorts, whereas the ΣDEP exposure distributions were fairly similar across cohorts.

**Figure 2:**
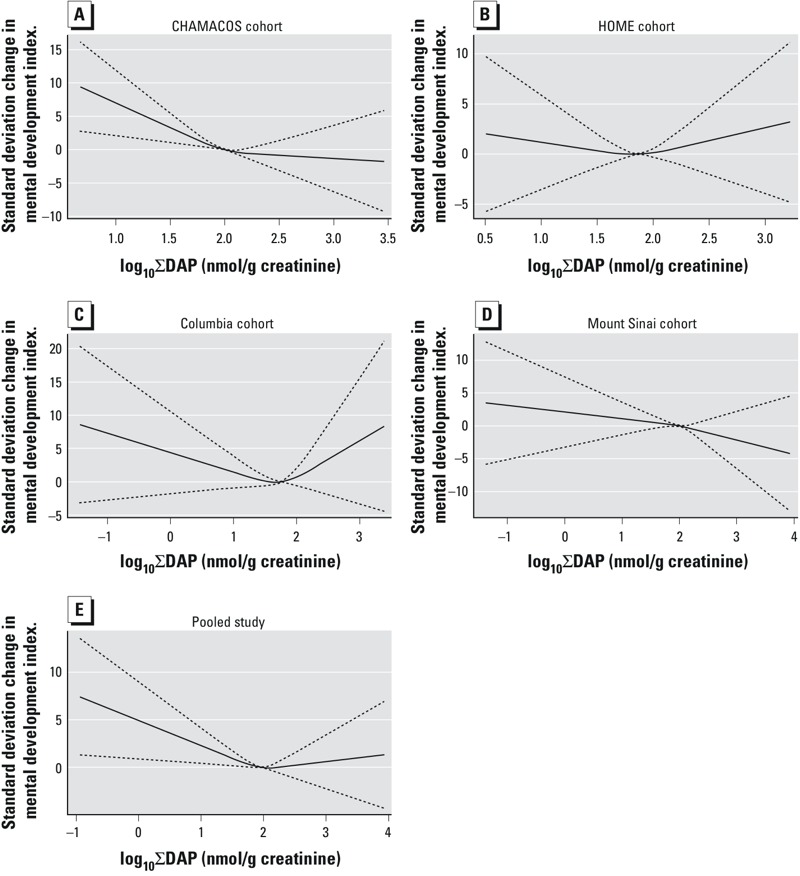
Restricted cubic splines for log_10_ ∑DAP association with the 24-month MDI in the (*A*) CHAMACOS cohort, (*B*) HOME cohort, (*C*) Columbia cohort, (*D*) Mount Sinai cohort, and (*E*) pooled population. Splines demonstrate a roughly linearly declining relationship in the individual cohorts and the overall pooled population below approximately 2 nmol/g creatinine, which attenuates at higher concentrations. Although the HOME (*B*) and Columbia (*C*) cohorts appear to show a U-shape curve, the 95% CIs (dashed lines) demonstrate substantial imprecision around these estimates.

***Twenty-four–month mental development index.*** The overall pooled dose–response function is consistent with a log_10_ linear term, although above approximately 2 nmol/g creatinine there is a leveling off, in large part due to a deviation in dose–response functions among cohorts, with HOME and Columbia demonstrating positive associations above that exposure level, whereas CHAMACOS and Mount Sinai both found negative associations. Nonetheless, a formal investigation of the suitability of a log_10_ linear term using a 1-df Wald chi-square test (α = 0.05) (Desquilbet and Mariotti 2010) did not reject linearity in the overall pooled exposure variable (Figure 2). All estimates are expressed per 10-fold increase in creatinine-corrected log_10_-transformed ΣDAP/DEP/DMP.

In a model adjusted for center, maternal education, marital status, maternal age at delivery, alcohol use during pregnancy, child sex, Caldwell Home environment score, and breastfeeding, there was significant heterogeneity by center in the association between ΣDMP and ΣDAP and the MDI (*p* = 0.05 and *p* = 0.09, respectively) (Table 3). CHAMACOS, Columbia, and Mount Sinai found negative associations between increasing ΣDAP exposure and the MDI, whereas the HOME study found a positive association. Only the CHAMACOS center-specific association reached statistical significance. In CHAMACOS, the MDI score at 24 months declined by 4.17 points per 10-fold increase in creatinine-corrected, log_10_ transformed ΣDAP (ΣDAP β = –4.17; 95% CI: –7.00, –1.33). The overall pooled association was also negative (ΣDAP β = –1.28; 95% CI: –2.58, 0.03), and stronger after adjustment for maternal race/ethnicity, smoking, and drug use during pregnancy (ΣDAP β = –1.48; 95% CI: –2.77, –0.19), although technically the inclusion of these covariates violates the positivity assumption. Nonetheless, significant heterogeneity in the center-specific associations limits the interpretability of the pooled estimates. This heterogeneity was driven in large part by the strong negative association in the CHAMACOS cohort. With the CHAMACOS population removed, the overall pooled association was attenuated (ΣDAP β = –0.71; 95% CI: –2.25, 0.83). Overall, the heterogeneity evident in the center-specific associations argues against interpreting the pooled association.

**Table 3:** Association of creatinine-corrected log_10_ prenatal OP metabolite levels and 24-month MDI and PDI considering heterogeneity by center.

Model	CHAMACOS [β per 10-fold increase (95% CI)]^*a*^	HOME [β per 10-fold increase (95% CI)]^*a*^	Columbia [β per 10-fold increase (95% CI)]^*a*^	Mount Sinai [β per 10-fold increase (95% CI)]^*a*^	Center-OP-interaction (*p*-value)^*a*^	Pooled [β per 10-fold increase (95% CI)]^*a*^	Pooled (extended model) [β per 10-fold increase (95% CI)]^*b*^
24-month MDI	*n *= 366	*n *= 257	*n *= 53	*n *= 225	*n *= 901	*n *= 901	*n *= 890
Log_10_ ∑DAP (nmol/gC)	–4.17 (–7.00, –1.33)	0.97 (–1.82, 3.76)	–0.79 (–4.17, 2.59)	–1.14 (–3.12, 0.84)	0.09	–1.28 (–2.58, 0.03)	–1.48 (–2.77, –0.19)
Log_10_ ∑DEP (nmol/gC)	–1.40 (–4.12, 1.32)	–1.10 (–3.42, 1.22)	0.11 (–2.88, 3.11)	–0.22 (–1.85, 1.42)	0.81	–0.57 (–1.69, 0.54)	–0.82 (–1.93, 0.28)
Log_10_ ∑DMP (nmol/gC)	–3.64 (–5.97, –1.32)	0.73 (–1.59, 3.04)	0.13 (–2.33, 2.58)	–0.80 (–2.52, 0.91)	0.05	–0.91 (–1.98, 0.16)	–1.06 (–2.12, 0.00)
24-month PDI	*n *= 368	*n *= 258	*n *= 55	*n *= 228	*n *= 909	*n *= 909	*n *= 898
Log_10_ ∑DAP (nmol/gC)	–1.35 (–4.10, 1.41)	1.09 (–1.63, 3.80)	–0.39 (–3.67, 2.90)	–0.27 (–2.17, 1.63)	0.67	–0.23 (–1.48, 1.03)	–0.08 (–1.34, 1.18)
Log_10_ ∑DEP (nmol/gC)	–0.91 (–3.54, 1.72)	–0.32 (–2.57, 1.93)	–0.35 (–3.13, 2.42)	0.52 (–1.03, 2.06)	0.79	–0.02 (–1.09, 1.04)	0.15 (–0.91, 1.22)
Log_10_ ∑DMP (nmol/gC)	–1.29 (–3.56, 0.97)	0.83 (–1.41, 3.08)	–0.42 (–2.79, 1.95)	–0.45 (–2.11, 1.21)	0.63	–0.35 (–1.39, 0.68)	–0.30 (–1.34, 0.73)
∑DAP = ∑DEP + ∑DMP. ^***a***^Models were adjusted for maternal education, marital status, and age at delivery, alcohol use during pregnancy, child sex, Home environment score quartile, and breastfeeding at least 3 months after birth, and include metabolite × center interaction terms. Tests for interaction were calculated using *F*-tests with 3 degrees of freedom. ^***b***^Additionally adjusted for race/ethnicity, smoking and drug use during pregnancy.							

We also explored heterogeneity in the pooled associations with MDI according to race/ethnicity without adjustment for center (Table 4). There was significant heterogeneity in the ΣDAP and ΣDMP associations with the MDI by race/ethnicity (*p* = 0.06 and *p* = 0.02, respectively), with the strongest negative associations found among Hispanics for ΣDAP and ΣDMP (ΣDAP β = –2.91; 95% CI: –4.71, –1.12; ΣDMP β = –2.34; 95% CI: –3.77, –0.91). The CHAMACOS population accounted for approximately 70% of all Hispanics included in this pooled analysis. Across racial/ethnic groups, the overall pooled association was still negative (ΣDAP β = –1.39; 95% CI: –2.67, –0.10), although this was to a large degree driven by the strong negative association among Hispanics.

**Table 4:** Association of creatinine-corrected log_10_ prenatal OP metabolite levels and 24-month MDI and PDI considering heterogeneity by race/ethnicity.

Model	White [β per 10-fold increase (95% CI)]^*a*^	Black [β per 10-fold increase (95% CI)]^*a*^	Hispanic [β per 10-fold increase (95% CI)]^*a*^	Race/ethnicity- OP interaction (*p*-value)^*a*^	Pooled [β per 10-fold increase (95% CI)]^*b*^
24-month MDI	*n *= 231	*n *= 142	*n *= 499	*n *= 872	*n *= 890
Log_10_ ∑DAP (nmol/gC)	–0.29 (–2.89, 2.31)	0.49 (–2.07, 3.05)	–2.91 (–4.71, –1.12)	0.06	–1.39 (–2.67, –0.10)
Log_10_ ∑DEP (nmol/gC)	–1.46 (–3.62, 0.69)	0.09 (–2.15, 2.33)	–1.00 (–2.57, 0.58)	0.60	–0.82 (–1.92, 0.29)
Log_10_ ∑DMP (nmol/gC)	–0.21 (–2.39, 1.98)	1.10 (–0.94, 3.14)	–2.34 (–3.77, –0.91)	0.02	–0.97 (–2.00, 0.07)
24-month PDI	*n *= 234	*n *= 144	*n *= 502	*n *= 880	*n *= 898
Log_10_ ∑DAP (nmol/gC)	2.34 (–0.21, 4.88)	–0.47 (–2.97, 2.03)	–0.41 (–2.18, 1.37)	0.18	0.26 (–1.00, 1.51)
Log_10_ ∑DEP (nmol/gC)	1.15 (–0.97, 3.27)	–0.57 (–2.75, 1.62)	0.27 (–1.24, 1.78)	0.54	0.32 (–0.75, 1.40)
Log_10_ ∑DMP (nmol/gC)	1.62 (–0.52, 3.75)	–0.12 (–2.15, 1.91)	–0.53 (–1.95, 0.89)	0.25	0.06 (–0.96, 1.08)
∑DAP**= ∑DEP + ∑DMP. ^***a***^Models were adjusted for maternal education, marital status and age at delivery, alcohol, drug use or smoking during pregnancy, child sex, quartiles of Home environment score, breastfeeding at least 3 months after birth, and birth before 2001, and include metabolite × race/ethnicity interaction terms. “Other” race/ethnicity was excluded due to small numbers. Tests for interaction were calculated using *F*-tests with 2 degrees of freedom. ^***b***^Pooled models include all race/ethnicity categories.					

We also examined heterogeneity of the associations with MDI by the child *PON1* –108C/T and *PON1* Q192R polymorphisms, in the whole population and within subgroups of race/ethnicity (Table 5). There was no significant heterogeneity attributable to the –108 C/T polymorphism in the whole population for the ΣDAP, ΣDEP, or ΣDMP metabolites. However, among whites there was significant heterogeneity in estimates by genotype category, albeit in an unexpected direction. Although the T allele is considered the at-risk allele, corresponding to lower PON1 enzyme activity, white carriers of the TT genotype had higher MDI scores with increasing ΣDAP exposure (ΣDAP β = 5.35; 95% CI: –0.41, 11.11), whereas the associations in the CC and CT genotype categories were inverse, and all confidence intervals included the null value. Among blacks and Hispanics, no significant heterogeneity by *PON1* –108C/T was detected. There was a significant interaction with the *Q192R* polymorphism overall for ΣDEP (*p* = 0.01), with inverse associations between exposure and MDI among carriers of the QR or QQ genotype, but not RR carriers. No significant heterogeneity was found overall for *PON1* Q192R and ΣDAP or ΣDMP. Among blacks, there was significant heterogeneity between ΣDAP and *PON1* Q192R (*p* = 0.08), although the confidence intervals were very imprecise. Inverse associations were found for carriers of the QR genotype, but not for the RR or QQ (at-risk) genotype.

**Table 5:** Association of creatinine-corrected log_10_ prenatal OP metabolite levels and 24-month MDI according to *PON1* genotype.

*PON1* genotype	Pooled				Blacks		Whites		Hispanics	
	*n*	Pooled β per 10-fold increase (95% CI) log_10_ ∑DAP (nmol/gC)^*a*^	Pooled β per 10-fold increase (95% CI) log_10_ ∑DEP (nmol/gC)^*a*^	Pooled β per 10-fold increase (95% CI) log_10_ ∑DMP (nmol/gC)^*a*^	*n*	β per 10-fold increase (95%CI) log_10_ ∑DAP (nmol/gC)^*b*^	*n*	β per 10-fold increase (95% CI) log_10_ ∑DAP (nmol/gC)^*b*^	*n*	β per 10-fold increase (95% CI) log_10_ ∑DAP (nmol/gC)^*b*^
–108 C/T	766				11		203		429
CC	280	–2.35 (–4.58, –0.12)	–0.61 (–2.55, 1.33)	–1.78 (–3.59, 0.03)	75	–0.50 (–4.33, 3.32)	58	–5.12 (–10.71, 0.48)	140	–2.31 (–5.95, 1.32)
CT	346	–0.12 (–2.22, 1.97)	–0.24 (–2.17, 1.69)	–0.45 (–2.17, 1.26)	37	3.47 (–1.74, 8.68)	96	–0.37 (–4.30, 3.56)	207	–1.55 (–4.43, 1.32)
TT (at risk)	140	–1.22 (–4.48, 2.03)	–1.74 (–4.56, 1.07)	0.14 (–2.34, 2.61)	7	–1.38 (–15.23, 12.47)	49	5.35 (–0.41, 11.11)	82	–5.54 (–9.67, –1.40)
*p*-Interaction		0.36	0.68	0.39		0.45		0.04		0.29
Q192R	762				112		206		429
RR	229	0.51 (–1.98, 3.01)	2.09 (–0.14, 4.31)	0.08 (–2.16, 2.32)	56	3.52 (–0.21, 7.26)	17	9.71 (–4.79, 24.22)	103	–3.86 (–7.97, 0.25)
QR	354	–2.31 (–4.60, –0.02)	–1.94 (–3.94, 0.07)	–1.23 (–2.86, 0.39)	47	–3.16 (–9.63, 3.32)	74	–4.10 (–8.69, 0.48)	224	–1.87 (–4.84, 1.09)
QQ (at risk)	179	–1.74 (–4.22, 0.75)	–1.93 (–4.10, 0.24)	–1.13 (–3.27, 1.02)	9	–11.85 (–25.51, 1.80)	115	1.15 (–2.53, 4.84)	102	–3.19 (–6.70, 0.33)
*p*-Interaction		0.23	0.01	0.62		0.04		0.08		0.71
∑DA*P *= ∑DEP + ∑DMP. ^***a***^Models were adjusted for maternal race/ethnicity, maternal education, age at delivery and marital status, smoking, alcohol or drug use during pregnancy, child sex, quartile of Home environment score, breastfeeding at least 3 months after birth, and birth before 2001, and include metabolite × genotype interaction terms. Tests for interaction were calculated using *F*-tests with 2 degrees of freedom. ^***b***^Models were adjusted for maternal education, age at delivery and marital status, smoking, alcohol or drug use during pregnancy, child sex, quartile of Home environment score, breastfeeding at least 3 months after birth, and birth prior to 2001, and include metabolite × genotype interaction terms. Tests for interaction were calculated using *F*-tests with 2 degrees of freedom.										

In sensitivity analyses, we examined whether there was significant heterogeneity (at *p* < 0.10) in the ΣDAP/ΣDEP/ΣDMP associations among Hispanics from different cohorts, among whites from different cohorts, and among blacks from different cohorts, and found none (data not shown). We also found that adjustment for a prenatal lead exposure biomarker did not substantially improve the fit (by AIC), nor did exclusion of lead from the model confound any associations of OP metabolites with the MDI (data not shown). And finally, we explored whether the overall fit of the pooled mixed effects model was improved with the addition of Bayley examiner and found no substantial difference in the main effect estimates compared with those that do not include Bayley examiner (data not shown).

***Twenty-four–month psychomotor development index.*** Associations between OP metabolites and the PDI were generally null at the 24-month visit, with no indication that any associations were nonlinear, and with no substantial heterogeneity by center (Table 3), race/ethnicity (Table 4), or *PON1* genotype (data not shown).

## Discussion

In this pooled analysis of four prospectively enrolled NIEHS/EPA-funded Children’s Environmental Health and Disease Prevention Research Centers, we estimated that each 10-fold increase in prenatal exposure to ΣDAPs is associated with an approximate 1-point decrease in the 24-month MDI, whether pooled across four cohorts without adjustment for race/ethnicity (β = –1.28; 95% CI: –2.58, 0.03), or pooled across race/ethnicities without adjustment for cohort (β = –1.48; 95% CI: –2.77, –0.19). Although the estimates in general lead to the same conclusion, they should both be interpreted with caution given that there was evidence of significant heterogeneity, particularly for the ΣDMP metabolites, that could in part be attributed to center and race/ethnicity, as well as a significant threat of residual confounding by factors exhibiting non-positivity across centers. As described above, non-positivity occurs when there is a lack of exposed and unexposed individuals at all levels of the observed confounders—in this case, differences in eligibility and enrollment criteria resulted in some centers that lacked study subjects at all levels of key covariates of concern (e.g., prenatal smoking and drug use, years of birth, language spoken in the home). We additionally found evidence of heterogeneity in associations by *PON1* genotype. In particular, we found evidence of heterogeneity in associations of ΣDAPs with the MDI according to *PON1* –108C/T genotype among whites, heterogeneity with ΣDEPs and MDI according to *PON1* Q192R in the whole population, and with ΣDAPs and *PON1* Q192R among both whites and blacks. There was no evidence of an association with PDI.

Although in some respects these cohorts were ideally suited for pooling because of their similar data collection tools for covariate and outcome data and biomarker measures obtained from the same laboratory, there are also differences among these cohorts that limit the interpretability of the overall pooled estimates. Eligibility and enrollment characteristics differed by center, as did the types of populations targeted (urban/suburban vs. agricultural) and the time frame of enrollment. Although some of the study-specific exclusions are unlikely to present a bias in the absence of a strong association between exposure and the characteristic in question (e.g., smoking and parity), other differences present more cause for concern (e.g., target population, enrollment year, gestational age at delivery exclusions). Mount Sinai excluded births < 32 weeks or < 1,500 g, CHAMACOS excluded births < 500 g, and Columbia measured exposure late in the third trimester of pregnancy, thus resulting in a truncated gestational age distribution that effectively excluded many preterm births. The Mount Sinai and Columbia populations individually found no associations between organophosphorus pesticide exposures and length of gestation (Berkowitz et al. 2004; Whyatt et al. 2005), but the HOME and CHAMACOS populations did, at least for some of the metabolites (Eskenazi et al. 2004; Rauch et al. 2012). Although these were relatively weak associations overall, still, to the extent that early delivery lies intermediate on the causal pathway between exposure and neurodevelopment, exclusions of very early deliveries may have to some extent biased estimates of associations. We believe, however, that any bias is likely to be small given that on a population level, births < 32 weeks gestation or < 1,500 g account for < 1% of singleton births in the United States (Martin et al. 2015). Of more significance are cohort differences in target population and enrollment year, which are sure to have a pronounced impact on the type and intensity of parent OP exposures.

Another challenge to pooling these four unique cohorts was in reconciling the underlying exposure biomarker distributions. The distribution of the ΣDAP biomarkers was severely right skewed. And although the ΣDEP biomarker concentrations were very similar across the cohorts, the median ΣDMP exposure biomarker concentrations were considerably higher in the CHAMACOS study than in the Mount Sinai (42% higher), HOME study (78% higher), and Columbia (256% higher) populations. The advantages of the log_10_ transformation in the presence of right skewed data include achieving a more normal distribution and reducing the impact of outliers in modeling. Moreover, the dose–response function in our pooled population was consistent with a log_10_ linear term. Applying a log_10_ transformation to these data results in the expansion of values at the low end of exposure and the constriction of values at the high end of exposure. For example, an increase in exposure concentration from 0.9 to 9.0 nmol/gC and from 9.0 to 90 nmol/gC both equate to a 1-log_10_ unit change. As a result, the absolute increase in exposure concentration implied by a change in log_10_ units is relative to where you are in the natural distribution. This feature comprises the major limitation of the log_10_ transformation—specifically, the interpretability of effect estimates in reference to an absolute change in exposure level is lost. This is particularly problematic when comparing across centers with dissimilar exposure distributions. Moreover, it is possible that to some extent our findings of center and race-driven heterogeneity in associations are in part a result of this transformation, in that the absolute change in biomarker concentration for the CHAMACOS study per log_10_ unit might be on average higher than the other centers, resulting in more elevated effect estimates in the CHAMACOS study.

Although DAP metabolites are the most commonly reported internal dosimeter of OP pesticide exposure, these biomarkers are nonspecific; the same metabolite can derive from multiple parent OPs that may differ in toxicity and potency. Therefore, a given DAP biomarker concentration from CHAMACOS, where subjects were exposed to pesticides used in agriculture as well as other sources of exposure (e.g., diet or residential use), may not mean the same thing as the same DAP biomarker concentration from the Mount Sinai or Columbia studies, which captured residential use of chlorpyrifos and diazinon during permissible periods, as well as dietary exposures. Although some parent compounds are used in both settings, others are not. It is, for example, plausible that the strong negative association observed between the dimethylphosphate metabolites and the MDI in the CHAMACOS population is attributable partly to local use of oxydemeton-methyl, a highly toxic OP solely used in agriculture. Unfortunately, our pooled data do not permit definitive identification of the parent compound mix to which a subject was exposed, and it is likely that the parent compound exposure composition is more similar across subjects within a given cohort than across cohorts.

Another important consideration is the relative contributions of parent compound exposures versus preformed DAP exposures. Direct exposure to parent OP pesticide compounds results in metabolism and the production of metabolites along with the biologically active oxon-form. However hydrolysis or photolysis of parent compounds present in food and the environment also generates preformed DAPs that are not toxic when consumed, and that are mostly excreted unchanged in urine (Chen L et al. 2012; Zhang et al. 2008). Thus, measurements of DAPs in urine may overestimate exposure to parent pesticide compounds.

Exposure to OP pesticides through the diet (Lu et al. 2005) may result in a higher fraction of preformed DAP exposure relative to parent compound exposure compared with exposure through direct OP pesticide applications (residentially or occupationally) or drift from nearby applications or para-occupational take-home exposures (Deziel et al. 2015; Quirós-Alcalá et al. 2012).

It is likely that the CHAMACOS population, located in an agricultural region with heavy continuing OP pesticide use, received a more substantial fraction of parent compound exposure relative to the Mount Sinai, Columbia, or HOME populations. Furthermore, the HOME population, which was enrolled after the U.S. EPA moved to restrict residential uses of OPs, likely experienced higher dietary versus environmental exposures compared with the two New York City cohorts, which both spanned periods of indoor use of chlorpyrifos and diazinon for cockroaches. These circumstances likely resulted in the HOME study population having the highest fraction of preformed DAP exposures out of their total DAP exposure, and therefore those with higher urinary DAP biomarkers may have eaten higher quality diets that are high in fruits and vegetables. These differences in source, route, and period may partly account for the significant center-driven heterogeneity that we identified in our analysis (*p* = 0.09), although there is a lack of empirical evidence to fully describe any differences in exposure source and intensity the participants in these cohorts experienced. Indeed, the pattern of associations across the centers appears to accurately reflect our *a priori* expectations of the magnitude of parent compound exposures these subjects were likely to have experienced. The CHAMACOS association was the strongest (β = –4.17; 95% CI: –7.00, –1.33), followed by very similar estimates between the Mount Sinai (β = –1.14; 95% CI: –3.12, 0.84) and Columbia populations (β = –0.79; 95% CI: –4.17, 2.59), with a positive association in the Cincinnati HOME study (β = 0.97; 95% CI: –1.82, 3.76). The CHAMACOS population, an agricultural population where 44% of the study subjects reported personally working in agriculture during pregnancy and 84% lived with farmworkers, is likely to have experienced the highest parent compound exposure burden. Mount Sinai and Columbia also were enrolled before the U.S. EPA action to limit residential exposures, so exposure to parent compounds was likely to have been similar between the two cohorts, although less than in the agriculturally exposed CHAMACOS cohort. And the HOME study, because of the geographic and sociodemographic groups enrolled and the time period of enrollment, was likely to have been exposed primarily through the diet, with the least direct parent compound exposure (Yolton et al. 2013). Therefore, although the same method of exposure assessment was employed in all four studies, the meaning of the biomarkers may differ across studies, so pooling may obscure, at least in part, important differences in associations across studies.

Additionally, DAP biomarkers of OP exposure have been shown to exhibit low reproducibility over the course of pregnancy in a subset of the Generation R study, a population-based cohort from the Netherlands, in which a major determinant of exposure level was found to be fruit intake (Spaan et al. 2015). Two of the cohorts included in this pooled analysis collected two urine samples during pregnancy (CHAMACOS and the HOME study), and the creatinine-corrected average of these two specimens was used, and two cohorts only collected urine samples in the third trimester. Classifying exposure based on the mean value in two samples (vs. one sample) is likely to reduce exposure misclassification due to high within-subject variability, but might obscure associations if effects are specific to a particular time window of exposure.

Other limitations of pooled analyses should be taken into consideration when weighing these results. Both confounder adjustment and an examination of heterogeneity in any OP associations by susceptibility factors were limited to covariates and characteristics that were shared by all centers. This may have resulted in differences between previously reported center-specific associations, undetected but important effect heterogeneity, or the possibility of residual confounding by factors, such as poverty, prenatal smoking, primary language used in the home, parity, and other factors, that could not be accounted for due to non-overlapping covariate distributions. Including such covariates into multivariable adjusted pooled models when the expected sample size for a given cohort is zero in that exposure-covariate strata violates our assumption of positivity (Cole and Hernán 2008)—that at every level of the confounders there are both exposed and unexposed participants. For example, additionally adjusting for race/ethnicity in a center-adjusted pooled model required that we assume that the effect of race is the same across each of the sites, an assumption that we could not explore for centers that lacked participants in one or more race/ethnicity group. Moreover, this assumption seemed tenuous given our knowledge of the differences in the CHAMACOS exposure source profile compared with all other cohorts. The analogous assumption is perhaps easier to accept for other covariates such as prenatal smoking or multiparity. We were able to assess differences only in our cohort-specific associations (with limited covariate adjustment that was consistent across all studies) from previously published estimates for the CHAMACOS (Eskenazi et al. 2007) and the Mount Sinai studies (Engel et al. 2011). For the CHAMACOS study, our current beta estimate for DAP metabolites and the MDI was slightly elevated relative to the prior paper, and there were essentially no differences for the other metabolites, or for any metabolite and the PDI. There were more differences in magnitude evident when comparing the Mount Sinai beta estimates, the current analysis yielding somewhat attenuated associations. Additionally, although pooling these cohorts affords us more power to investigate gene–environment interactions, our power was still limited, particularly when stratified by race/ethnicity, to estimate stable associations in the rare genotype categories.

In conclusion, although it is important to determine whether there is an overall effect of OP exposure experienced in diverse settings on child neurodevelopment both for policy and for research purposes, a careful examination of the site-specific findings remains essential in understanding the implications of heterogeneity in exposure period, source, and susceptibility.

## Supplemental Material

(202 KB) PDF

## References

[r1] Barr DB, Allen R, Olsson AO, Bravo R, Caltabiano LM, Montesano A (2005). Concentrations of selective metabolites of organophosphorus pesticides in the United States population.. Environ Res.

[r2] Barr DB, Wong LY, Bravo R, Weerasekera G, Odetokun M, Restrepo P (2011). Urinary concentrations of dialkylphosphate metabolites of organophosphorus pesticides: National Health and Nutrition Examination Survey 1999–2004.. Int J Environ Res Public Health.

[r3] Bayley N (1993). Bayley Scales of Infant Development. 2nd ed..

[r4] Berkowitz GS Obel J Deych E Lapinski R Godbold J Liu Z 2003 Exposure to indoor pesticides during pregnancy in a multiethnic, urban cohort. Environ Health Perspect 111 79 84, doi:10.1289/ehp.5619 12515682 PMC1241309

[r5] Berkowitz GS Wetmur JG Birman-Deych E Obel J Lapinski RH Godbold JH 2004 *In utero* pesticide exposure, maternal paraoxonase activity, and head circumference. Environ Health Perspect 112 388 391, doi:10.1289/ehp.6414 14998758 PMC1241872

[r6] Bravo R, Caltabiano LM, Weerasekera G, Whitehead RD, Fernandez C, Needham LL (2004). Measurement of dialkyl phosphate metabolites of organophosphorus pesticides in human urine using lyophilization with gas chromatography-tandem mass spectrometry and isotope dilution quantification.. J Expo Anal Environ Epidemiol.

[r7] Caldwell BM, Bradley RH (1984). Administration Manual: Home Observation For Measurement of the Environment. Revised ed..

[r8] Chen A Yolton K Rauch SA Webster GM Hornung R Sjödin A 2014 Prenatal polybrominated diphenyl ether exposures and neurodevelopment in U.S. children through 5 years of age: the HOME Study. Environ Health Perspect 122 856 862, doi:10.1289/ehp.1307562 24870060 PMC4123029

[r9] Chen J, Chan W, Wallenstein S, Berkowitz G, Wetmur JG (2005). Haplotype-phenotype relationships of paraoxonase-1.. Cancer Epidemiol Biomarkers Prev.

[r10] Chen J Kumar M Chan W Berkowitz G Wetmur JG 2003 Increased influence of genetic variation on PON1 activity in neonates. Environ Health Perspect 111 1403 1409, doi:10.1289/ehp.6105 12928148 PMC1241633

[r11] Chen L, Zhao T, Pan C, Ross JH, Krieger RI (2012). Preformed biomarkers including dialkylphosphates (DAPs) in produce may confound biomonitoring in pesticide exposure and risk assessment.. J Agric Food Chem.

[r12] Cole SR, Hernán MA (2008). Constructing inverse probability weights for marginal structural models.. Am J Epidemiol.

[r13] Costa LG, Richter RJ, Li WF, Cole T, Guizzetti M, Furlong CE (2003). Paraoxonase (PON 1) as a biomarker of susceptibility for organophosphate toxicity.. Biomarkers.

[r14] Curl CL, Fenske RA, Kissel JC, Shirai JH, Moate TF, Griffith W (2002). Evaluation of take-home organophosphorus pesticide exposure among agricultural workers and their children.. Environ Health Perspect.

[r15] Desquilbet L, Mariotti F (2010). Dose-response analyses using restricted cubic spline functions in public health research.. Stat Med.

[r16] Deziel NC Friesen MC Hoppin JA Hines CJ Thomas K Freeman LE 2015 A review of nonoccupational pathways for pesticide exposure in women living in agricultural areas. Environ Health Perspect 123 515 524, doi:10.1289/ehp.1408273 25636067 PMC4455586

[r17] Engel SM, Berkowitz GS, Barr DB, Teitelbaum SL, Siskind J, Meisel SJ (2007). Prenatal organophosphate metabolite and organochlorine levels and performance on the Brazelton Neonatal Behavioral Assessment Scale in a multiethnic pregnancy cohort.. Am J Epidemiol.

[r18] Engel SM Wetmur J Chen J Zhu C Barr DB Canfield RL 2011 Prenatal exposure to organophosphates, paraoxonase 1, and cognitive development in childhood. Environ Health Perspect 119 1182 1188, doi:10.1289/ehp.1003183 21507778 PMC3237356

[r19] Eskenazi B Harley K Bradman A Weltzien E Jewell NP Barr DB 2004 Association of *in utero* organophosphate pesticide exposure and fetal growth and length of gestation in an agricultural population. Environ Health Perspect 112 1116 1124, doi:10.1289/ehp.6789 15238287 PMC1247387

[r20] Eskenazi B Marks AR Bradman A Harley K Barr DB Johnson C 2007 Organophosphate pesticide exposure and neurodevelopment in young Mexican-American children. Environ Health Perspect 115 792 798, doi:10.1289/ehp.9828 17520070 PMC1867968

[r21] Fukuto TR (1990). Mechanism of action of organophosphorus and carbamate insecticides.. Environ Health Perspect.

[r22] Grube A, Donaldson D, Kiely T, Wu L (2011). Pesticides Industry Sales and Usage: 2006 and 2007 Market Estimates. EPA 733-R-11-001..

[r23] Holland N Furlong C Bastaki M Richter R Bradman A Huen K 2006 Paraoxonase polymorphisms, haplotypes, and enzyme activity in Latino mothers and newborns. Environ Health Perspect 114 985 991, doi:10.1289/ehp.8540 16835048 PMC1513322

[r24] Icenogle LM, Christopher NC, Blackwelder WP, Caldwell DP, Qiao D, Seidler FJ (2004). Behavioral alterations in adolescent and adult rats caused by a brief subtoxic exposure to chlorpyrifos during neurulation.. Neurotoxicol Teratol.

[r25] Levin ED, Addy N, Baruah A, Elias A, Christopher NC, Seidler FJ (2002). Prenatal chlorpyrifos exposure in rats causes persistent behavioral alterations.. Neurotoxicol Teratol.

[r26] Lu C, Bravo R, Caltabiano LM, Irish RM, Weerasekera G, Barr DB (2005). The presence of dialkylphosphates in fresh fruit juices: implication for organophosphorus pesticide exposure and risk assessments.. J Toxicol Environ Health A.

[r27] Martin JA, Hamilton BE, Osterman MJK, Curtin SC, Mathews TJ (2015). Births: Final Data for 2013.. Natl Vital Stat Rep.

[r28] Quirós-Alcalá L, Bradman A, Smith K, Weerasekera G, Odetokun M, Barr DB (2012). Organophosphorous pesticide breakdown products in house dust and children’s urine.. J Expo Sci Environ Epidemiol.

[r29] Rauch SA Braun JM Barr DB Calafat AM Khoury J Montesano AM 2012 Associations of prenatal exposure to organophosphate pesticide metabolites with gestational age and birth weight. Environ Health Perspect 120 1055 1060, doi:10.1289/ehp.1104615 22476135 PMC3404666

[r30] Rauh VA, Garfinkel R, Perera FP, Andrews HF, Hoepner L, Barr DB (2006). Impact of prenatal chlorpyrifos exposure on neurodevelopment in the first 3 years of life among inner-city children.. Pediatrics.

[r31] Ray DE, Richards PG (2001). The potential for toxic effects of chronic, low-dose exposure to organophosphates.. Toxicol Lett.

[r32] Ricceri L, Markina N, Valanzano A, Fortuna S, Cometa MF, Meneguz A (2003). Developmental exposure to chlorpyrifos alters reactivity to environmental and social cues in adolescent mice.. Toxicol Appl Pharmacol.

[r33] Spaan S, Pronk A, Koch HM, Jusko TA, Jaddoe VW, Shaw PA (2015). Reliability of concentrations of organophosphate pesticide metabolites in serial urine specimens from pregnancy in the Generation R Study.. J Expo Sci Environ Epidemiol.

[r34] Timofeeva OA, Roegge CS, Seidler FJ, Slotkin TA, Levin ED (2008). Persistent cognitive alterations in rats after early postnatal exposure to low doses of the organophosphate pesticide, diazinon.. Neurotoxicol Teratol.

[r35] U.S. EPA (U.S. Environmental Protection Agency) (2000). Chlorpyrifos. Revised Risk Assessment and Agreement with Registrants..

[r36] U.S. EPA (2001). Diazinon Revised Risk Assessment and Agreement with Registrants..

[r37] Vatanparast J, Naseh M, Baniasadi M, Haghdoost-Yazdi H (2013). Developmental exposure to chlorpyrifos and diazinon differentially affect passive avoidance performance and nitric oxide synthase-containing neurons in the basolateral complex of the amygdala.. Brain Res.

[r38] Westreich D, Cole SR (2010). Invited commentary: positivity in practice.. Am J Epidemiol.

[r39] Wetmur JG, Kumar M, Zhang L, Palomeque C, Wallenstein S, Chen J (2005). Molecular haplotyping by linking emulsion PCR: analysis of paraoxonase 1 haplotypes and phenotypes.. Nucleic Acids Res.

[r40] Whyatt RM, Camann D, Perera FP, Rauh VA, Tang D, Kinney PL (2005). Biomarkers in assessing residential insecticide exposures during pregnancy and effects on fetal growth.. Toxicol Appl Pharmacol.

[r41] Whyatt RM Rauh V Barr DB Camann DE Andrews HF Garfinkel R 2004 Prenatal insecticide exposures and birth weight and length among an urban minority cohort. Environ Health Perspect 112 1125 1132, doi:10.1289/ehp.6641 15238288 PMC1247388

[r42] Yolton K Xu Y Sucharew H Succop P Altaye M Popelar A 2013 Impact of low-level gestational exposure to organophosphate pesticides on neurobehavior in early infancy: a prospective study. Environ Health 12 1 79, doi:10.1186/1476-069X-12-79 24034442 PMC3848803

[r43] Zhang X, Driver JH, Li Y, Ross JH, Krieger RI (2008). Dialkylphosphates (DAPs) in fruits and vegetables may confound biomonitoring in organophosphorus insecticide exposure and risk assessment.. J Agric Food Chem.

