# Supplementary material for: Prenatal Organophosphorus Pesticide Exposure and Child Neurodevelopment at 24 Months: An Analysis of Four Birth Cohorts
Source: Environ Health Perspect. 2015 Sep 29;124(6):822–30. doi: 10.1289/ehp.1409474 (PMC4892910; doi:10.1289/ehp.1409474)
Supplement: (202 KB) PDF [file ehp.1409474.s001.acco.pdf]

Environ Health Perspect
DOI: 10.1289/ehp.1409474

Note to Readers: *EHP* strives to ensure that all journal content is accessible to all readers. However, some figures and Supplemental Material published in *EHP* articles may not conform to 508 standards due to the complexity of the information being presented. If you need assistance accessing journal content, please contact ehp508@niehs.nih.gov. Our staff will work with you to assess and meet your accessibility needs within 3 working days.

Supplemental Material

Prenatal Organophosphorus Pesticide Exposure and Child Neurodevelopment at 24 Months: An Analysis of Four Birth Cohorts

Stephanie M. Engel, Asa Bradman, Mary S. Wolff, Virginia A. Rauh, Kim G. Harley, Jenny H. Yang, Lori A. Hoepner, Dana Boyd Barr, Kimberly Yolton, Michelle G. Vedar, Yingying Xu, Richard W. Hornung, James G. Wetmur, Jia Chen, Nina T. Holland, Frederica P. Perera, Robin M. Whyatt, Bruce P. Lanphear, and Brenda Eskenazi

Table of Contents

Table S1. Exposure Distributions of Individual Dialkylphosphate Metabolites by Center and Measurement Period

Table S2. Distributions of Dialkylphosphate Metabolite Sums in Individual Cohorts

Table S1. Exposure Distributions of Individual Dialkylphosphate Metabolites by Center and Measurement Period

First Trimester Spot Urine	CHAMACOS (n = 375)			HOME (n = 262)								
	LOD	% <LOD[a]	N Missing due to analytic interference	LOD	% <LOD[a]	N missing due to analytic interference						
Diethylphosphate	0.2 - 0.8	36.0%	2	0.6	30.2%	0						
Diethylthiophosphate	0.1 - 0.6	45.9%	2	0.4	22.5%	0						
Diethyldithiophosphate	0.1 - 0.3	36.3%	0	0.4	58.0%	0						
Dimethylphosphate	0.6 - 1.2	41.9%	2	0.6	27.5%	0						
Dimethylthiophosphate	0.2 - 1.1	27.7%	0	0.2	13.0%	0						
Dimethyldithiophosphate	0.1 - 1.0	45.3%	3	0.5	37.4%	0						
**Third Trimester Spot Urine**	**CHAMACOS (n = 355)**			**HOME (n = 258)**			**Columbia (n = 63)**			**Mount Sinai (n = 234)**		
	**LOD**	**% <LOD[a]**	**N Missing due to analytic interference**	**LOD**	**% <LOD[a]**	**N Missing due to analytic interference**	**LOD**	**% <LOD**	**N Missing due to analytic interference**	**LOD**	**% <LOD**	**N Missing due to analytic interference**
Diethylphosphate	0.2	42.3%	0	0.6	41.9%	0	0.3	41.3%	0	0.3	61.5%	7
Diethylthiophosphate	0.1	0.9%	0	0.4	38.0%	0	0.4	33.3%	0	0.4	21.4%	0
Diethyldithiophosphate	0.1	73.5%	0	0.4	78.7%	0	0.1	52.4%	0	0.2	92.7%	2
Dimethylphosphate	0.6	5.4%	0	0.6	44.2%	0	0.5	44.4%	0	0.5	46.6%	0
Dimethylthiophosphate	0.2	0.8%	0	0.2	10.9%	0	0.7	50.8%	0	0.4	11.1%	2
Dimethyldithiophosphate	0.1	41.7%	3	0.5	54.3%	0	0.2	68.3%	0	0.3	78.6%	0

[a] This column only includes values below the LOD when instrument read values were unavailable. CHAMACOS and HOME received some instrument read values below the LOD. For these studies, values below the LOD were only imputed if instrument read values were unavailable.

Table S2. Distributions of Dialkylphosphate Metabolite Sums in Individual Cohorts

**Distributions of Dialkylphosphate Metabolite Sums**	**Geometric Mean (GSD) (nmol/gC)**	**Min nmol/gC**	**25% nmol/gC**	**50% nmol/gC**	**75% nmol/gC**	**Max nmol/gC**
CHAMACOS (n =377)						
Total Dialkylphosphate (∑DAP)	111.70 (2.75)	4.69	56.02	107.54	225.58	3003.04
Total Diethylphosphate (∑DEP)	15.94 (2.89)	0.15	7.78	16.35	33.90	319.89
Total Dimethylphosphate (∑DMP)	71.58 (3.47)	0.64	32.89	78.34	168.21	2999.54
HOME (n = 265)						
Total Dialkylphosphate (∑DAP)	76.17 (3.41)	2.32	34.19	79.52	181.21	1715.39
Total Diethylphosphate (∑DEP)	11.16 (4.52)	0.05	4.65	14.18	34.44	199.07
Total Dimethylphosphate (∑DMP)	44.19 (4.50)	0.07	16.94	44.00	117.96	1686.76
Columbia (n = 60)						
Total Dialkylphosphate (∑DAP)	39.16 (9.87)	0.04	17.69	57.25	146.23	2567.63
Total Diethylphosphate (∑DEP)	7.77 (23.53)	0.00	5.58	20.03	49.39	231.80
Total Dimethylphosphate (∑DMP)	4.39 (134.50)	0.00	2.16	22.02	118.48	2564.99
Mount Sinai (n = 234)						
Total Dialkylphosphate (∑DAP)	73.80 (6.49)	0.04	29.47	95.94	209.03	8498.60
Total Diethylphosphate (∑DEP)	11.46 (11.58)	0.00	6.93	18.22	56.88	670.42
Total Dimethylphosphate (∑DMP)	40.09 (9.87)	0.00	13.82	55.17	167.88	8498.46
